# Reconstructing a B-Cell Clonal Lineage. II. Mutation, Selection, and Affinity Maturation

**DOI:** 10.3389/fimmu.2014.00170

**Published:** 2014-04-22

**Authors:** Thomas B. Kepler, Supriya Munshaw, Kevin Wiehe, Ruijun Zhang, Jae-Sung Yu, Christopher W. Woods, Thomas N. Denny, Georgia D. Tomaras, S. Munir Alam, M. Anthony Moody, Garnett Kelsoe, Hua-Xin Liao, Barton F. Haynes

**Affiliations:** ^1^Department of Microbiology, Boston University School of Medicine, Boston, MA, USA; ^2^Department of Mathematics and Statistics, Boston University, Boston, MA, USA; ^3^Center for Viral Hepatitis Research, Johns Hopkins University, Baltimore, MD, USA; ^4^Duke Human Vaccine Institute, Duke University Medical Center, Durham, NC, USA; ^5^Department of Medicine, Duke University Medical Center, Durham, NC, USA; ^6^Department of Pathology, Duke University Medical Center, Durham, NC, USA; ^7^Hubert-Yeargan Center for Global Health, Duke University Medical Center, Durham, NC, USA; ^8^Department of Pediatrics, Duke University Medical Center, Durham, NC, USA; ^9^Department of Immunology, Duke University Medical Center, Durham, NC, USA

**Keywords:** somatic hypermutation, experimental influenza infection, antibody selection, antibody affinity maturation, phylogenetics

## Abstract

Affinity maturation of the antibody response is a fundamental process in adaptive immunity during which B-cells activated by infection or vaccination undergo rapid proliferation accompanied by the acquisition of point mutations in their rearranged immunoglobulin (Ig) genes and selection for increased affinity for the eliciting antigen. The rate of somatic hypermutation at any position within an Ig gene is known to depend strongly on the local DNA sequence, and Ig genes have region-specific codon biases that influence the local mutation rate within the gene resulting in increased differential mutability in the regions that encode the antigen-binding domains. We have isolated a set of clonally related natural Ig heavy chain–light chain pairs from an experimentally infected influenza patient, inferred the unmutated ancestral rearrangements and the maturation intermediates, and synthesized all the antibodies using recombinant methods. The lineage exhibits a remarkably uniform rate of improvement of the effective affinity to influenza hemagglutinin (HA) over evolutionary time, increasing 1000-fold overall from the unmutated ancestor to the best of the observed antibodies. Furthermore, analysis of selection reveals that selection and mutation bias were concordant even at the level of maturation to a single antigen. Substantial improvement in affinity to HA occurred along mutationally preferred paths in sequence space and was thus strongly facilitated by the underlying local codon biases.

## Introduction

B-cells that respond to infection or vaccination are induced by signaling through their B-cell receptors to proliferate and differentiate into plasmacytes and memory cells. Short-lived plasmacytes secrete antibody and provide immediate protection from the eliciting agent; memory cells and long-lived plasmacytes persist clonally for very long times, providing protection against recurring challenges from the same or closely related agents (1). Cells that go on to find persistent clones are subject to affinity maturation in their post-exposure development. During affinity maturation, the affinity of the B-cell receptor for antigens on the eliciting agent is substantially increased, resulting in a more potent response on recall (2).

Affinity maturation proceeds through somatic hypermutation, the introduction of point mutations into the rearranged immunoglobulin (Ig) genes that encode the B-cell receptor. Those B-cells that thereby acquire an increased affinity for the antigen gain a proliferative advantage and come to dominate the activated B-cell population. Affinity maturation is crucial for humoral immune protection, conferring greater neutralization capacity (3) and opsonization efficiency (4), and is generally correlated with higher vaccine efficacy (5). In fact, lack of effective affinity maturation has been directly implicated in adverse outcomes for at least one vaccine (6).

The rate of somatic hypermutation at a given position with an Ig variable region is significantly influenced by the local DNA sequence – both the nucleotide at that position and sequence of nucleotides containing it (7). Codon usage in Ig V-gene segments is strongly biased, with zones of high mutability largely overlapping with the complementarity-determining regions (CDR), which encode the antibody’s antigen-binding residues (8, 9). Thus, somatic mutation drives Ig genes along statistically favored paths through the genotype space. Some combinations of substitutions will therefore be visited much more rapidly than others involving the same number of changes. Each Ig gene segment has been involved in the response to a huge number of antigens over the course of its evolutionary history and has experienced selection pressure to enhance its role as a template for affinity maturation.

Technology for the isolation of native heavy-chain/light-chain pairs and their subsequent recombinant synthesis have recently been developed (10, 11) and refined (12), making it feasible to determine the biophysical properties of large numbers of monoclonal antibodies (mAb). We have complemented this technology with the development of computational tools that substantially improve our ability to infer the unmutated common ancestor of a set of clonally related antibodies, and the corresponding maturation intermediates (13).

We have now applied these methods to the detailed study of the maturation pathways of a B-cell clone whose antibody genes were isolated from a human experimental influenza infection study, providing an elucidation of the interplay of mutational constraints and selection on antigen-binding affinity. One of our aims in this study is to examine the influence that this differential mutability has on a specific instance of affinity maturation to a given antigen: the immune response to influenza hemagglutinin (HA) in a human subject. This question clearly goes beyond the issue of codon bias as a statistical regularity to inquire about influence of codon bias in a specific case. The relationship between these two questions is analogous to the phenomenon of HCDR3 length in autoimmune disease. There one has the statistical observation that B-cells with long HCDR3 are counter-selected during development (14), yet the role of long HCDR3 for individual autoantibodies is rarely understood. In our case, we know that the mutation frequency is higher on average in regions that encode amino acids that are more likely, on average, to contact epitopes. In this study, we examine the interplay of differential mutation frequency and selection in the evolution of a single antibody lineage.

Specifically, we demonstrate that intraclonal affinity maturation proceeded by stepwise accumulation of affinity-enhancing mutations and that mutation and selection interacted synergistically. These insights and others gained by application of the tools we have developed promise to facilitate the effective harnessing of affinity maturation for vaccine engineering.

## Results

### Isolation and identification of anti-influenza hemagglutinin a B-cell clone CL2569

Human subjects were experimentally infected intranasally with influenza virus (15). Eighty-six natural heavy-chain/light-chain gene pairs were isolated from one subject (subject EI13) on day 4 after exposure. Among these, we found three clonally related sets. Two of the clones contained two antibodies each; the other contained five. The members of this five-member clone, designated CL2569, all bind HA in the *K*_d_ = 1–20 nM range. Four of these antibodies are of the IgM isotype while the other is IgA1. The light chain in each antibody is Ig kappa. The remainder of this study describes our analysis of CL2569.

The antibodies are highly diversified. The heavy chains have a mean (±SD) pairwise difference of 28.0 ± 5.4 nucleotides (nt) and 16.7 ± 3.6 amino acids (aa); the light chains have an average pairwise difference of 18.0 ± 2.5 nt and 8.2 ± 1.5 aa.

We inferred the unmutated ancestor (UA) and intermediates along the affinity maturation pathways by computing the Bayesian posterior probability mass function on nucleotide states at each position of the heavy and light chains conditioned by the data and the maximum-likelihood phylogram as described in the companion study (13). The mutations acquired along each branch were enumerated and classified according to the IMGT classification (16) (Table 1). The UA and all intermediates for both heavy and light chains were synthesized using the same recombinant technology used to synthesize the observed antibodies.

**Table 1:** **Classification of mutations in CL2569 heavy- and light-chain histories**.

	Heavy chains		Light chains	
	Non	Synon	Non	Synon
FR	40	20	14	7
CDR	17	11	11	4

*FR, framework region; CDR, complementarity-determining region; Non, non-synonymous; Synon, synonymous*.

**Figure 1:**
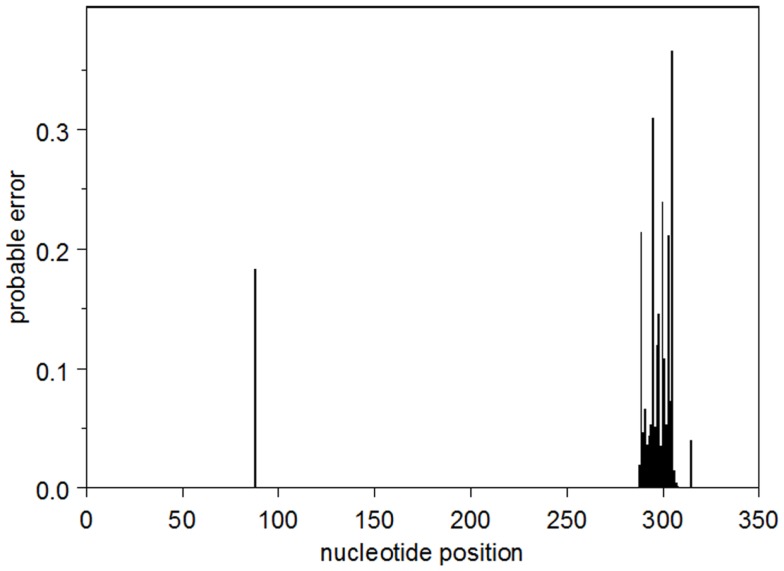
**The profile of the probable error in the modal heavy chain UA**.

The probable error profile for the heavy-chain UA is shown in Figure 1. Briefly, the sum of the probable errors over all positions is 3.2. There are five nucleotide positions where the marginal posterior probability of the modal nucleotide is <0.8, all of which occur in CDR3. Importantly, at these somewhat lower-confidence positions, the inferred modal CDR3 is identical to all five observed sequences. The summed probable errors for each of the inferred intermediates is less than that of the inferred UA and decreases as one gets closer to the observed sequences. The kappa chain UA is known with high confidence. The sum of the probable errors is 0.33.

### The dissociation constant decreases exponentially with uniform rate over the duration of the process

**Figure 2:**
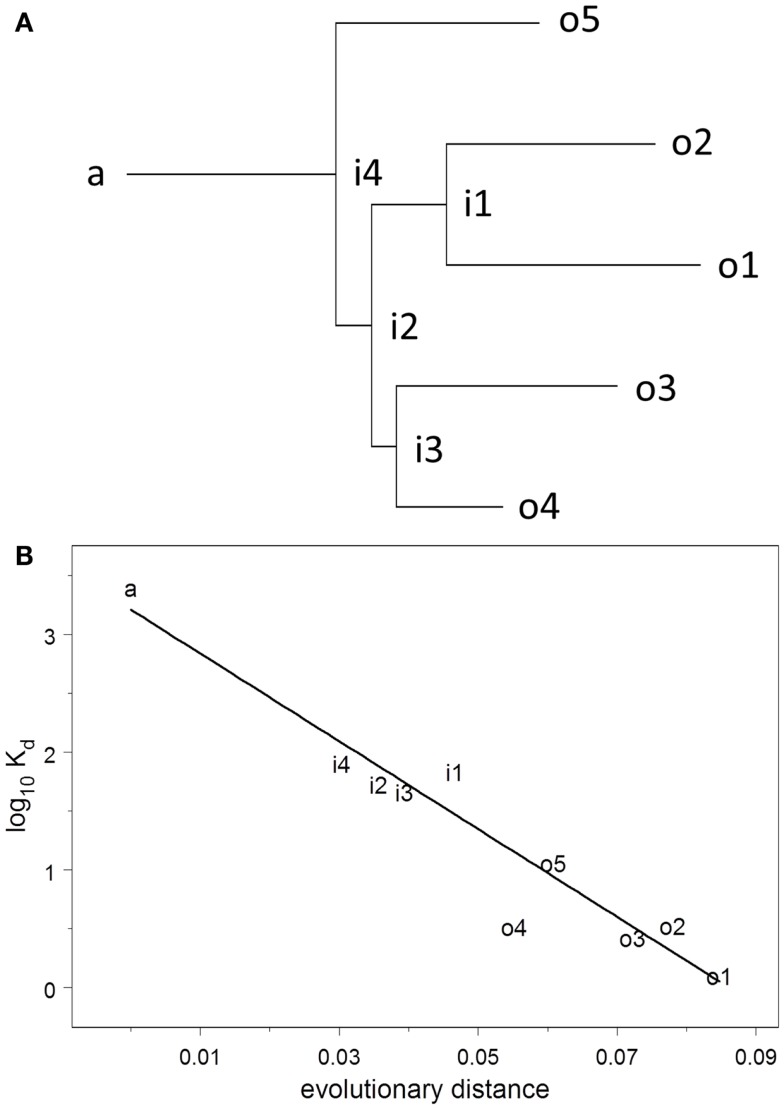
**(A)** Maximum-likelihood clonal tree showing observed (o), intermediate (i), and ancestral (a) sequences. The tree was inferred using both heavy and light chains. **(B)** Regression analysis of log_10_ *K*_d_ vs. evolutionary distance.

The dissociation constant *K*_d_ for binding to HA of the Brisbane strain of influenza virus was measured using ELISA on solutions of monoclonal antibody prepared at known concentrations. *K*_d_ was estimated by non-linear curve fitting simultaneously on all data for each plate. The UA binds to HA very weakly but measurably, *K*_d_ = 2.6 μM. Throughout the evolutionary process, *K*_d_ declines uniformly and exponentially (*R*^2^ = 0.92), falling 50–74% (95% confidence interval) for each 1% increase in evolutionary distance (Figure 2). The affinities of the observed antibodies are approximately three orders of magnitude higher than that of the ancestor, an improvement that occurs over a total evolutionary distance of 6–9% nucleotide differences.

### Interaction between selection and mutability

To gage the force of selection in molecular evolution, deviations in the ratio of the number of synonymous mutations to the number of non-synonymous mutations from that expected under the null hypothesis of selection-free evolution are often used for statistical testing (17). For antibody somatic evolution, mutations are further classified by region, occurring in the CDR or framework regions (FR) and various combinations specific deviations from expected values within these classifications used in statistical tests [see, e.g., Ref. (18)]. Crucially, the distribution expected under the no selection null hypothesis for Ig somatic evolution is not trivially computed. Because the codon bias has been adapted for Ig plasticity, empirical estimation of the distributions under the null cannot be avoided.

The model we use to estimate parameters and perform tests is straightforwardly derived using likelihood-based methods in statistics. We nevertheless describe the model in some detail below so that the argument may be essentially self-contained.

In order to explore the interplay of selection and mutability, we use a non-linear regression model and multiple independent categorical distributions^1^ in which every gene position along each branch of the clonal tree can either be unmutated, mutated synonymously, or mutated non-synonymously. That is, there are three possible classifications for each nucleotide, and the “mutation type” variable takes one of the two values: $T\in \left\{S,N\right\}$. For the *i*th nucleotide in gene *g*, the variable $x_{\text{gi}}^{T}$ is an indicator for the mutation type. For example, if the nucleotide in question has been mutated non-synonymously along the branch leading up to *g* from its parent sequence *a*(*g*), we have $x_{\text{gi}}^{N}=1$ and $x_{\text{gi}}^{S}=0$. If the nucleotide is not mutated at all, we have $x_{\text{gi}}^{N}=0$ and $x_{\text{gi}}^{S}=0$.

The relevant likelihood function is the product of independent categorical distributions, whose log (we work with the log of the likelihood function for convenience) is

(1) logL=∑gi∑TxgiT log PagiT+1−xgi•log1−Pagi•

where $P_{a\left(g\right)i}^{T}$ is the probability that the *i*th nucleotide in the parent of gene *g* would have mutation type *T*. The dot in place of an index indicates summation over that index, for example, $1-P_{a\left(g\right)i}^{•}$ is the probability that the nucleotide in question is not mutated. It is the dependency of these probabilities on the covariates that we model.

The covariates are themselves properties of the specific nucleotide expressed in terms of probabilities. There is first the probability that a given nucleotide mutates at all. This probability is the product of the sequence-specific mutation rate μ_ai_ and the effective evolutionary time τ along the relevant branch. Then, we have the probability $\sigma_{\text{ai}}^{T}$ that a mutation occurring at the position and gene in question will have type *T* (that is, conditional on there being a mutation at all). This probability depends on the codon in which the nucleotide is found and its position within the codon. But it also depends on the local sequence (19); these influences have to be estimated for the nucleotide at each position of the gene. Finally, there is the impact of selection. Once a mutation has occurred, it must survive to fixation in order to be observed.

The covariates we will consider for predicting the survival of mutations at the *i*th position of gene *a* are the type *T* of the mutation, the region $R\left(i\right)\in \left\{\text{FR,CDR}\right\}$ that contains position *i*, and the mutability μ_ai_. Note that the dependence of the survival probability on mutability is over and above the role of the mutability in inducing the mutation initially. Indeed, it is the dependence of survival on mutability that is of primary concern for this study. The dependence of survival probability on region is given by the terms $\gamma_{R}^{T}$. The ratios of these terms give the relative survival probabilities. Because they are introduced as multiplicative rather than additive effects, they are subject, without loss of generality, to the multiplicative constraint $\gamma_{\text{FR}}^{\text{Syn}}\gamma_{\text{CDR}}^{\text{Syn}}\gamma_{\text{FR}}^{\text{Non}}\gamma_{\text{CDR}}^{\text{Non}}=1$.

Combining all the component probabilities then gives the probability that gene *g* has acquired an observed mutation of type *T* at position *i* and has survived. It is given by

(2) $$P_{\text{gi}}^{T}=\tau \mu_{a\left(g\right)i}\sigma_{a\left(g\right)i}^{T}\left(\gamma_{R(i)}^{T}+\beta_{T}\mu_{a(g)i}\right).$$

The local sequence specificity of μ_ai_ and $\sigma_{\text{ai}}^{T}$ are estimated using external data as described in the supplementary information.

For each hypothesis being tested, we impose the specific constraints on the model parameters in Eq. 2 that correspond to the hypothesis, estimate the remaining parameters by maximizing the likelihood. We then test hypotheses using the likelihood ratio test (20) where applicable, and compare models using the Akaike information criterion (AIC). The AIC is a penalized likelihood, appropriate for model selection where the likelihood ratio test is inapplicable because the respective models are not nested (21).

Local mutability is strongly informative. We compare two models: in the first (Model 0), the mutability is constant over positions μ*_i_* = μ*_j_* for all positions *i* and *j*. In the second (Model 1), the mutability is determined by the local sequence μ*_i_* = *m_j_* where *m_i_* is the mutability for the local sequence context at position *i*, estimated from an independent dataset (see Materials and Methods section). For this test, assume that selection is based on the covariate region × type, and allow $\gamma_{R}^{T}$ to vary subject to the multiplicative constraint above, whereas β*_T_* = 0 for both *T*. The models are not nested, so we use AIC and relative likelihood for the comparison. The model with empirical mutability is substantially better supported by the data than is the constant-mutability model (relative likelihood = 3 × 10^8^).

Region × type is informative in selection. If region and type are used to classify each potential mutation into one of the four classes that are then used to model the selection process, the predictive power of the model is increased. On comparing the selection-free null model with empirical local mutability (Model 1) with the alternative model in which $\gamma_{R}^{T}$ are fit to the data (Model 2: β*_T_* = 0, μ*_i_* = *m_i_*), we reject the null model (likelihood ratio test, *p* = 0.014).

Mutability × type is informative in selection. In addition to the mutability that is used to predict the generation of mutations, we may use mutability as a covariate for predicting selection. The resulting model has both linear and quadratic terms in the mutability. On comparing the null model that recognizes type, but not region (Model 3: $\gamma_{\text{FR}}^{T}=\gamma_{\text{CDR}}^{T}$, β*_T_* = 0 and μ*_i_* = *m_i_*), with the alternative model in which β*_T_* are fit to the data (Model 4: $\gamma_{\text{FR}}^{T}=\gamma_{\text{CDR}}^{T}$ μ*_i_* = *m_i_*), we reject the null model (likelihood ratio test, *p* = 0.010).

Mutability × type is slightly more informative than region × type in selection. Both region × type and mutability × type have been shown to be predictive. To determine which covariate is more effective as a predictor, we perform a model comparison by AIC; comparing the region × type model (Model 2) with the mutability × type model (Model 4). Both have four degrees of freedom, so by AIC, the comparison favors the mutability × type model (relative likelihood = 1.35).

**Figure 3:**
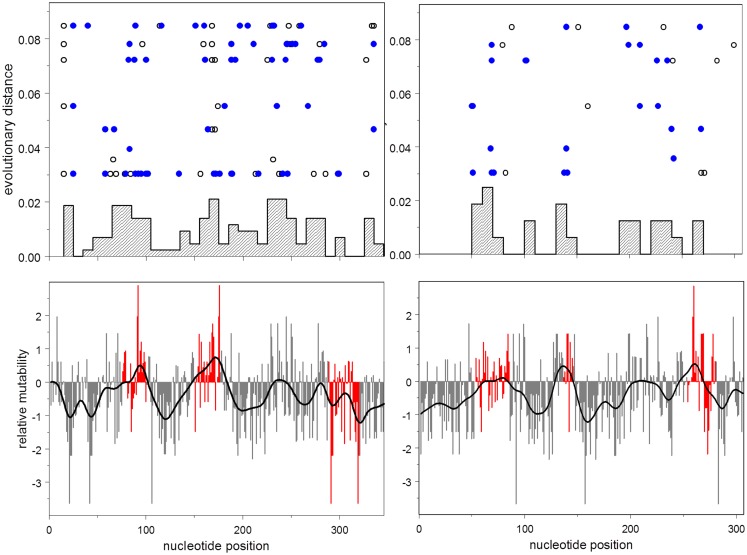
**Lower: mutability by position for heavy (left) and light (right) chains**. Mutability at CDR nucleotides is shown in red. Upper: histogram by position of accumulated non-synonymous mutations; evolutionary distance vs. position for each synonymous (open disks), and non-synonymous (closed disks) mutation.

This result is illustrated in Figure 3, which shows the distribution of relative mutabilities in relation to region and the distribution of observed non-synonymous mutations over both gene position and evolutionary time.

The AIC-optimal model uses both mutability × type and region × type to predict mutations. Given the covariates to which we have access, the largest model has μ*_i_* = *m_i_*, and both $\gamma_{R}^{T}$ and β*_T_* are free to vary. This model (Model 5) has the minimum AIC of all models, and all those models that are nested within it are rejected by likelihood ratio tests (*p* < 0.05). The coefficients of the optimal model are shown in Table 2.

**Table 2:** **Maximum-likelihood estimates for the coefficients in the optimal model**.

Model	Mutability	$\gamma_{\text{FR}}^{\text{Syn}}$	$\gamma_{\text{CDR}}^{\text{Syn}}$	$\gamma_{\text{FR}}^{\text{Non}}$	$\gamma_{\text{CDR}}^{\text{Non}}$	β_Syn_	β_Non_	AIC
0	Constant	(1)	(1)	(1)	(1)	(0)	(0)	640.0
1	Empirical	(1)	(1)	(1)	(1)	(0)	(0)	589.2
2	Empirical	0.75	2.12	0.67	(0.94)	(0)	(0)	584.6
3	Empirical	1.18	(1.18)	(0.85)	(0.85)	(0)	(0)	589.2
4	Empirical	2.25	(2.25)	(0.44)	(0.44)	−25.8	16.6	584.0
5	Empirical	1.58	3.23	0.34	(0.58)	−21.4	13.8	579.0
6	Empirical	1.29	(1.29)	(0.78)	(0.78)	9.63	(9.63)	589.0

*Parentheses indicate that the parameter is invariant at the indicated value in the model considered*.

The selection observed is predominantly purifying. Having determined that selection is measurably occurring, we investigate the nature of the selection by examining the coefficients of the model fit (Table 2). In both CDR and FR, the coefficients for non-synonymous mutations are significantly smaller than those for synonymous mutations, consistent with a scenario in which deleterious mutations were introduced in cells that did not survive selection.

Mutability × type is more informative than mutability alone. We have shown that mutability × type is informative. An informative test, the meaning of which will be elaborated on in the discussion, is whether the contribution of mutability to the survival of a mutation depends on the mutation type. For this comparison, we take the null model (Model 6) to have β_FR_ = β_CDR_ and $\gamma_{\text{FR}}^{T}=\gamma_{\text{CDR}}^{T}$, and the alternative model (Model 4) with β*_T_* free to vary. The null model is rejected (likelihood ratio test, *p* = 8 × 10^−3^).

It is crucial here to understand that this last test is a test of whether type (synonymous vs. non-synonymous) interacts in the statistical sense with mutability (the evolved biases in the targeting of somatic hypermutation) to influence the probability that a mutation survives to fixation. It is taken as given that type alone does influence a mutation’s survival probability. It is further taken as given that mutability alone influences whether a mutation occurs in the first place or not. This test is a test of whether mutability is informative regarding the probability that a mutation survives selection. Selection cannot act on synonymous mutations, so evidence that mutability is correlated with selective survival must come from examination of the interaction term between mutability and type. This interaction term is equivalent to β_FR_ − β_CDR_. The rejected null hypothesis is that this quantity is zero.

## Discussion

In this study, by inference and expression of the UA and inferred intermediate antibodies of a single clone, we have directly demonstrated the stepwise maturation of antibodies. Such stepwise maturation has been assumed on theoretical grounds (22), but the technology to observe it has not been utilized before now.

The antibodies of clone CL2569 bind influenza HA and are highly mutated. For these reasons, they almost certainly represent a secondary response. In fact, the most likely scenario for the ontogeny of this lineage is that it was formed via affinity maturation during an earlier infection or vaccination and was subsequently activated into differentiation to plasmacytes by the experimental infection without undergoing further affinity maturation. The subject was infected with the H3N2 A/Wisconsin/67/2005 strain of influenza virus; preliminary binding assays were done on HA from several strains including the infecting strain, H1 A/Brisbane/59/2007, and several others. Although the maturation patterns were similar across several of the strains, the affinities measured against H1 A/Brisbane/59/2007 were generally higher (15). The infection study was performed in 2008, so previous infection in the subject with influenza strains circulating in 2007 is consistent with this observed reactivity to H1 A/Brisbane/59/2007.

The recovered mAb in this clonal lineage were mostly IgM with a single member that was IgA1, and all the members had a degree of somatic hypermutation consistent with one or more prior rounds of antigen-driven germinal center maturation. Recent work by Pape et al. (23) has shown that in mice IgM-memory B-cells and class-switched memory B-cells have different circulation kinetics, such that IgM-memory B-cells persist after class-switched memory B-cells have disappeared from circulation. Furthermore, upon restimulation with antigen, IgM-memory B-cells were less likely to produce a secondary response in the presence of antigen-specific plasma antibody. Thus, it is interesting that the members of this clonal lineage bind to various previously circulating strains including the older H3 A/Johannesburg/33/1994 strain, that the antibodies were predominantly IgM, were hypermutated, and did not significantly contribute to the plasma antibody pool 4 weeks after experimental infection (15). All these findings suggest that this lineage is an example of such an IgM-memory B-cell clone isolated from an influenza-infected human subject.

Like other Darwinian processes, affinity maturation arises in the interplay between the generation of diversity and the subsequent selection of fitter variants. Affinity maturation, however, is a somatic process; properties of the germline gene segments that facilitate efficient maturation are preserved for the next germline generation (8). Thus, mutation and selection in affinity maturation are very strongly intertwined with mutations that are more likely on average to confer advantage, produced more frequently than those that are more likely on average to confer disadvantage. This circumstance has a practical consequence, complicating the analysis of selective pressure. We have overcome that problem by estimating the relevant characteristics of somatic hypermutation from a collection of human heavy chain genes rearranged out of frame and insusceptible to selection.

### Selection and mutability synergize during affinity maturation to HA

**Figure 4:**
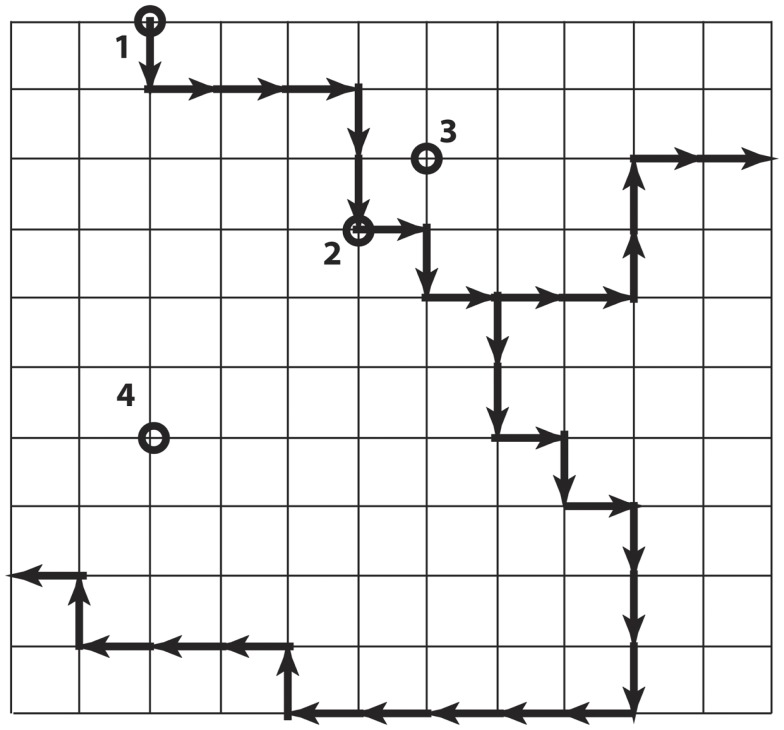
**Simplified illustration of genotype space with preferred directions**. Each node is a DNA sequence, and neighbors differ by one nucleotide. The dark arrows show preferred directions, meaning the mutation along the direction of the arrow occurs at a higher rate than mutations along the regular paths. The nodes labeled 2, 3, and 4 are all six steps from node 1, but differ in the number of non-preferred steps that must be taken to arrive there from 1.

The local codon bias that is present in Ig V-gene segments and increases mutability in the CDR creates a strongly non-uniform probability distribution over the links between Ig genes in the genotype space (Figure 4). Each of the Ig genes at the nodes of this space has an effective affinity for the antigen HA associated with it, which presumably determines the relative fitness of B-cells expressing the antibody encoded by that gene. Because of the mutational bias, from any starting node there are preferred nodes, which are visited with greater probability and in less time on average, than others. The question addressed here is whether the sequences more likely to be visited during somatic hypermutation because of this bias are also more likely to encode antibodies that confer a selective advantage.

Figure 4 is a simple cartoon intended to illustrate the idea. The grid represents the genotype space (although the topology is not at all realistic). The dark arrows indicate the directions of preferred mutations. We consider the node 1 to be the starting node. The other nodes 2–4 are each six mutations away from node 1, but they differ in the number of non-preferred mutations that are required to reach them. In the real system, we can estimate the mutation rate for each link, and in particular can estimate the mutation rates over the links connecting the nodes actually occupied during affinity maturation. We also have measured the affinity at each of these nodes, and know that they represent increases over time. So the question is, “are the visited nodes largely close to the preferred paths (as are nodes 2 and 3 in Figure 4), or randomly placed with respect to the preferred paths (illustrated by node 4)?”

We expect that such correlation between mutational preference and selective advantage holds on average over the history of antigens encountered by the gene segment in question. It is hypothesized that this is the reason why such local codon bias exists in the first place. The question addressed here is whether such a correlation exists, not on average, but in this particular instance, for this one specific antigen.

The mutability is defined at each nucleotide position as the probability of a mutation at that position conditional on there being exactly one mutation in the gene, and no selection on the gene product. In the presence of selection, the probability that a mutation will be fixed is the product of the probability that the mutation occurs at all and the probability that, once it has occurred, it is preserved through selection. The hypothesis we are testing is that the second of these probabilities, the probability of preservation, is itself functionally depending on the mutability. The order of the causality would be that the mutabilities have been adjusted, largely through codon usage, to make evolution toward the potentially advantageous genes more rapidly and more reliably.

**Figure 5:**
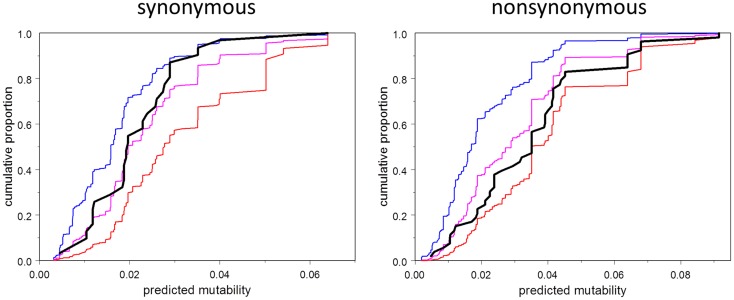
**Cumulative distribution function (CDF) of mutability among observed mutations (black), and corresponding to three models: order 0 (no effect of mutability at all, blue), order 1 (consistent with selection random with respect to mutability, magenta), second order (selection proportional to mutability, red)**. Note that the observed CDF for synonymous mutations is approximately consistent with the order one model, and falls between the order zero and order one curves in any case. The CDF for non-synonymous mutations falls between the order one and order two curves.

We address the question in Figure 5, which shows the empirical cumulative distribution plots of synonymous and non-synonymous mutations as a function of mutability, compared to three theoretical models: zero order (mutability has no influence, even on the probability of having a mutation in the first place), first order (mutability has the influence expected under selection-free conditions), and second-order (probability of selection is directly proportional to mutability). The plots show that the synonymous mutations are consistent, as expected, with the first-order model. Indeed, this plot should be regarded as a test of the accuracy of the estimated mutability, which appears to be adequate, although the mutabilities of the higher-mutability positions may be somewhat over-estimated. In contrast, the observed non-synonymous mutations fall between the first- and second-order curves, consistent with synergy between local codon bias and selection. Figure 5 is merely suggestive; the direct test of the relevant hypothesis (Model 4 vs. Model 6) provides stronger evidence. This test says that the influence of mutability on the survival of a mutation depends on the type of mutation, whether synonymous or non-synonymous. If the mutation is non-synonymous, the mutability has greater positive predictive power than that of synonymous type.

## Conclusion

Strikingly, despite the fact that the dissociation constant changed by three orders of magnitude from the common ancestor to the observed mature antibodies, the distribution of mutations is heavily biased toward those with high intrinsic mutability, suggesting that selection worked in synergy with local codon bias in the maturation of CL2569. This analysis suggests that affinity maturation is strongly constrained to occur by mutational diffusion along preferred paths in genotype space, with selection acting negatively on genotypes in this network that fail to confer enhanced antigen-binding affinity. There is no evidence for selection pulling the evolving clone substantially out of the mutationally preferred paths.

There are many highly effective vaccines that work through the induction of a potent humoral response, but there are many devastating infectious diseases for which no effective vaccine is yet available in spite of intense research efforts, including malaria, hepatitis C, and HIV-1. The agents of these diseases do not typically elicit protective natural immunity, so new approaches to vaccine development may be indicated. One such approach is predicated on the observation that the efficiency of immunogen stimulation of germinal center naïve and intermediate B-cell antibodies is determined by immunogen affinity for B-cell precursor B-cell receptor (24–26). Design of immunogens with high-affinity binding for antibody UAs and their intermediates is now possible with the computational methods described in this study (27). It is our hope that the emerging understanding of the intertwined mechanisms of diversification and selection in affinity maturation will open new avenues for vaccine engineering.

## Materials and Methods

### Statistical and computational

All analyses and computational manipulations were performed using software developed in the Kepler laboratory.

#### ELISA data analysis

The data from the ELISA dilution series were fit to a Hill function with Hill coefficient = 1 and additive background (28). The maximum value of the optical density and the value of the background optical density were taken to be equal over all wells on a given plate.

#### Inference of unobserved antibodies: ancestral rearrangement and maturation intermediates

We compute the posterior probability mass function on the nucleotides at each position of the unmutated common ancestor given the set of clonally related observed Ig genes of CL2569, as described in detail in Ref. (13).

#### Inference of somatic hypermutation sequence specificity

We searched NCBI Genbank for rearranged human Ig heavy-chain variable-region genes and retrieved and validated 34,546 genes. We eliminated genes with possible clonal relatives in the set by randomly eliminating all but one of each sequence within groups likely to be clonally related. Two antibodies were considered likely to be related if they shared the same inferred IGHV and IGHJ genes (without regard to allele) and shared at least 75% nucleotide identity in CDR3^2^. From these, we selected those that were likely to have been rearranged out of frame as evidenced by the number of nucleotides between the intact invariant cysteine in VH FR 3 and the intact invariant tryptophan in JH being other than a multiple of three.

By counting frame-shift mutations in the VH-encoded part of the gene, which have resulted from somatic mutations or sequencing error, we estimate the likely number of genes that would have frame-shift mutations in CDR3 to be about 195 genes. That is about 11% of our candidate non-productively rearranged genes are likely to have been rearranged in-frame and to have acquired their frame-shift mutations subsequently.

To ameliorate the impact this contamination could have on the downstream analysis, we removed all genes inferred to have been rearranged to a VH1 family member. The reason for this filtering step is that the positions of pentanucleotides in the remaining sequences will be significantly de-correlated from the positions of the corresponding pentanucleotides in the target sequences, which are rearranged to a VH1 family member.

After this filtering step, 1707 sequences remained, containing 9961 nucleotide substitutions in 423,654 total bases.

The mutation frequency for the central position at each pentanucleotide motif was computed by scanning each inferred UA. Of the 4^5^ (1024) possible pentanucleotides, 938 motifs were present in the total dataset, 922 in the out of frame dataset. Of the motifs with at least 100 observations among the UAs in the non-productive set, 24 of them had no mutations. In contrast, the motif AGCTA, which is consistent with the canonical “hot-spot” RGYW, was mutated at the center position 112 out of 618 times for a frequency of 18%.

For comparison to other such datasets previously assembled, we also computed the trinucleotide mutation frequencies. The spearman correlation between our trinucleotide mutation frequencies and the corresponding mutability indices from unselected sequences in the study by Shapiro et al. (29) is 0.80, indicating a high level of agreement between the two sets.

Rather than use, the raw count ratios for the mutability and mutation spectrum estimates directly (which is likely to result in over- or under-fitting), we chose to fit these data to a variable-motif length model using regression trees. The first statistical treatment of sequence specificity in somatic mutation produced hot-spot motifs of different lengths (7) and it seems natural to fit such a model now that much more data are available.

The end result of this estimation procedure is a set of nucleotide motifs that are mutually exclusive and complete (every nucleotide in any DNA sequence will belong to exactly one motif) to each member of which is assigned a mutation rate. Each motif may be up to 5 nt long. The procedure is as follows.

Each node in the decision tree contains a pentanucleotide motif of the form *n*_1_, *n*_2_, *n*_3_, *n*_4_, *n*_5_ in which each *n_i_* = {A, G, T, C, R, Y, S, W, N} where R, Y, S, W, N are the IUPAC symbols respectively for purine (A or G), pyrimidine (T or C), weak (A or T), strong (G or C), and any (A, G, T, or C) nucleotide.

The function to be maximized, the objective function, is the log of the marginal likelihood summed over all nodes in the tree. The overall likelihood is the product of the binomial likelihoods at each node. At each node, the prior distribution on mutations is a beta distribution with parameters α = 1, β = 47. The beta distribution is chosen because it is conjugate to the binomial distribution, and the specific parameters are chosen because they maximize the information entropy at the observed average mutation frequency in the set, 2.1%. As such, this prior is the most uninformative prior consistent with the average mutation frequency.

The marginal likelihood for a node with *m* mutations and *u* unmutated bases is computed by integrating over the mutation probabilities in the product of the likelihood and prior density functions giving:

(3) $$L_{\text{MU}}\left(m,u|\alpha ,\beta \right)=\frac{\Gamma \left(\alpha +m\right)\Gamma \left(\beta +u\right)\Gamma \left(\alpha +\beta +1\right)}{\Gamma \left(\alpha \right)\Gamma \left(\beta \right)\Gamma \left(\alpha +\beta +m+u+1\right)}$$

where Γ is the gamma function.

The tree-building algorithm is greedy, choosing the best available split at each node. Allowed splits at any step in the algorithm at any single position in the motif are as follows:

$$\begin{array}{llll} & N\to R∕Y,N\to S∕W,R\to A∕G,Y\to T∕C,\; & & \;\\ & \;S\to G∕C,W\to A∕T.\; & & \; \end{array}$$

This scheme ensures that each pentanucleotide is mapped to exactly one terminal node on the tree at all stages of the procedure. A node is declared terminal if the product of the marginal likelihoods for the two daughter nodes in the optimal split is less than the marginal likelihood of the parent node, that is, if the likelihood cannot be increased any further at that node.

The result of applying this process to our count data is a tree with 55 terminal nodes. Among these, the one with the lowest relative mutability is with YTGGS with posterior mean $\hat{p}=7.9\times 10^{-4}$. The AID “hot-spot” motif AGCT is assigned to the NAGCW node, with $\hat{p}=8.8\times 10^{-2}$.

#### Regression model for the dependence of selection on mutability

The model scheme for analysis of selection is described in the main text. The data used are all nucleotide position in the heavy-chain variable regions up to and including the nucleotides of the FR3 invariant cysteine codon. The fitting of parameters by maximum likelihood was performed by numerical optimization using the Nelder–Mead simplex algorithm using a software implementation based largely on that described in Numerical Recipes (30).

Statistical hypothesis tests are based on the likelihood ratio test when models are nested. Model comparison is done by differential AIC expressed as relative likelihoods (31).

### Experimental

#### Clinical protocol

The clinical EI protocol study was performed at Retroscreen Virology Ltd. (Brentwood, UK) as previously described (32) using a protocol approved by their local ethics board and the Duke IRB. Subjects were prescreened and provided informed consent before being given a nasal challenge with influenza A/Wisconsin/67/2005 (H3N2) challenge stock manufactured under current good manufacturing practices by Baxter BioScience (Vienna, Austria). Intranasal challenge was given using 10^3.08^ TCID50 to subject EI13 from whom the antibodies described in this study were derived. In this protocol, blood was drawn before challenge, then daily on days 0–7, and on day 28 after challenge. Symptoms were recorded twice daily using a modified Jackson scoring system (33). Productive infection was confirmed by active viral shedding detected by assays of nasal washes obtained during the 7-day quarantine period.

#### Single-cell flow cytometry sorting strategy

Human peripheral blood mononuclear cell samples collected 7 days after infection with A/Wisconsin/67/2005 (H3N2) were labeled with panels of fluorochrome-antibody conjugates specific for human CD3 (PE-Cy5), CD16 (PE-Cy5), CD19 (APC-Cy7), CD20 (PE-Cy7), CD27 (Pacific Blue), CD235a (PE-Cy5), IgD (PE), IgM (FITC) (all, BD Biosciences, San Jose, CA, USA), CD14 (PE-Cy5), and CD38 (APC-Cy5.5) (both Invitrogen, Carlsbad, CA, USA). Plasma cells/plasmablasts were sorted into 20 μl/well RT/PCR buffer in 96-well plates as described (10, 12) by gating on CD3^−^ CD14^−^ CD16^−^ CD235a^−^ CD19^ +^ CD20^−/lo^ CD27^hi^ CD38^hi^ cells. All antibody reagents were tittered and used at optimal concentrations for flow cytometry.

#### PCR amplification of plasmablast/plasma cell immunoglobulin VH and VL variable-region genes

The Ig VH and VL variable-region genes of the sorted plasmablast were amplified by RT and nested PCR using the method as reported (11). The PCR products amplified by this method contain enough coding region sequences for the constant regions of either heavy- or light-chain genes for allowing the identification of IgH subclass and light-chain types (12). Isolated VH and VL variable-region genes were used to assemble full-length Ig IgG1 heavy- and light-chain expression cassette by overlapping to express recombinant IgG1 antibodies using the method as described (12).

#### Expression of VH and VL variable-region genes as IgG1 recombinant mAb

The isolated Ig VH and VL gene pairs were assembled by PCR into the linear full-length Ig heavy- and light-chain gene expression cassettes for production of recombinant mAbs by transfection in the human embryonic kidney cell line, 293T (ATCC, Manassas, VA, USA) using the methods as described (12). The purified PCR products of the paired Ig heavy- and light-chain gene expression cassettes were co-transfected into near confluent 293T cells grown in 6-well (2 μg of DNA for each cassettes per well) tissue culture plates (Becton Dickson, Franklin Lakes, NJ, USA) using PolyFect (Qiagen, Valencia, CA, USA) or Effectene (Qiagen Valencia, CA, USA) using protocols recommended by the manufacturers. Six to eight hours after transfection, the 293T cells were fed with fresh culture medium supplemented with 2% FCS and were incubated at 37°C in a 5% CO_2_ incubator. Culture supernatants were harvested 3 days after transfection and quantified for expressed IgG levels and screened for antibody specificity.

Antibodies that bound HA in a screening assay as well as the inferred UA and intermediate clonal antibodies were produced on a larger scale so that screening assays could be replicated and broadened to more fully define the range of binding activity of expressed plasma cell derived-antibodies. Purified recombinant antibodies were produced in bulk cultures by transient transfection using Ig heavy- and light-chain genes cloned in pcDNA plasmids (12). The Ig heavy- and light-chain gene expression cassettes used for production of recombinant antibodies for initial screening were cloned into pcDNA 3.3 (Invitrogen, Carlsbad, CA, USA) for production of purified recombinant mAbs using standard molecular protocol, and co-transfected into 293T cells cultured in T175 flasks using PolyFect (Qiagen, Valencia, CA, USA) or polyethylenimine (34), cultured in DMEM supplemented with 2% FCS. Recombinant mAbs were purified from culture supernatants of the transfected-293T cells using anti-human Ig heavy-chain-specific antibody–agarose beads (Sigma, St. Louis, MO, USA) using the method as previously described (12, 34). Purified antibodies used in the study were confirmed having typical patterns of predominant whole IgG in SDS-PAGE and Western blots under reducing and non-reducing conditions (12).

#### Binding antibody multiplex assay

Concentration of recombinant mAbs secreted in the transfected-293T cell culture in the supernatants was determined using a method previously described (12). The expressed recombinant mAb were assayed for antibody reactivity by a standardized binding antibody multiplex assay (35) performed in a GCLP compliant manner. Binding specificities to influenza vaccine 2007 (Fluzone^®^ 2007), trivalent influenza vaccine 2008 (Fluzone^®^ 2008), and baculovirus-derived HA proteins (H1N1 A/Brisbane/59/2007, H1N1 A/California/04/2009, H1N1 A/Solomon Islands/03/06, H3N2 A/Brisbane/10/2007, H3N2 A/Johannesburg/33/1994, H3N2 A/Johannesburg/33/1994, H3N2 A/Wisconsin/67/05, B/Florida/04/06; Protein Sciences, Meriden, CT, USA) were determined using purified mAb diluted serially starting at 50 μg/ml.

#### ELISA data analysis for estimation of *K*_d_

Purified mAb prepared at known concentrations were evaluated by ELISA against baculovirus-expressed purified hemagglutinin (H1 A/Brisbane/59/2007; Protein Sciences, Meriden, CT, USA). Samples were diluted serially for the analysis and data were analyzed using the model

(4) $$y_{i}=\log\left[\alpha +\left(\beta -\alpha \right)\frac{c_{i}}{K_{\text{d}}+c_{i}}\right]+\varepsilon_{i}$$

where *y_i_* is the log of the optical density measured at the *i*th dilution, α is the background optical density, β is the maximum optical density, *K*_d_ is the equilibrium dissociation constant, *c_i_* is the known concentration of analyte at the *i*th dilution, and the ε are independent, identically distributed Gaussian errors. For each antibody studied, the parameters of this model were fit using software developed for the purpose (28).

## Conflict of Interest Statement

The authors declare that the research was conducted in the absence of any commercial or financial relationships that could be construed as a potential conflict of interest.

## Supplementary Material

The Supplementary Material for this article can be found online at http://www.frontiersin.org/Journal/10.3389/fimmu.2014.00170/abstract

Datasheet 1 **Sequence alignment for CL2569 heavy chain, including observed and inferred sequences**.

Datasheet 2 **Sequence alignment for CL2569 light chain, including observed and inferred sequences**.

Datasheet 3 **Tables of results for sequence specificity of mutation frequency**.

## Footnotes

^1^Such a product distribution is similar to a multinomial distribution, but has different probabilities at each site.

^2^This is admittedly a crude estimation procedure, but sequence set is small enough that we expect few if any errors from its use. Furthermore, we are unconcerned about falsely excluding unrelated sequences, which is the only likely error to be made by this method. Finally, the proper statistical procedure for testing clonal relatedness is sufficiently complex (Thomas B. Kepler, in preparation) that to put aside the space in a paper that does not require its full power would be distracting.
